# A method for direct estimation of left ventricular global longitudinal strain rate from echocardiograms

**DOI:** 10.1038/s41598-022-06878-1

**Published:** 2022-03-07

**Authors:** Brett A. Meyers, Melissa C. Brindise, Shelby Kutty, Pavlos P. Vlachos

**Affiliations:** 1 School of Mechanical Engineering, Purdue University, 585 Purdue Mall, West Lafayette, IN 47907 USA; 2 Weldon School of Biomedical Engineering, Purdue University, 206 S. Martin Jischke Dr., West Lafayette, IN 47907 USA; 3 Department of Mechanical Engineering, Pennsylvania State University, University Park, PA 16802 USA; 4 Taussig Heart Center, Johns Hopkins University School of Medicine, Baltimore, MD 21287 USA

**Keywords:** Cardiology, Techniques and instrumentation, Biological physics

## Abstract

We present a new method for measuring global longitudinal strain and global longitudinal strain rate from 2D echocardiograms using a logarithmic-transform correlation (LTC) method. Traditional echocardiography strain analysis depends on user inputs and chamber segmentation, which yield increased measurement variability. In contrast, our approach is automated and does not require cardiac chamber segmentation and regularization, thus eliminating these issues. The algorithm was benchmarked against two conventional strain analysis methods using synthetic left ventricle ultrasound images. Measurement error was assessed as a function of contrast-to-noise ratio (CNR) using mean absolute error and root-mean-square error. LTC showed better agreement to the ground truth strain ${({\varvec{R}}}^{2}=0.91)$ and ground truth strain rate ${({\varvec{R}}}^{2}=0.85)$ compared with agreement to ground truth for two block-matching speckle tracking algorithms (one based on sum of square difference and the other on Fourier transform correlation; strain ${({\varvec{R}}}^{2}=0.70)$, strain rate ${({\varvec{R}}}^{2}=0.70)$). A 200% increase in strain measurement accuracy was observed compared to the conventional algorithms. Subsequently, we tested the method using a 53-subject clinical cohort (20 subjects diseased with cardiomyopathy, 33 healthy controls). Our method distinguished between normal and abnormal left ventricular function with an AUC = 0.89, a 5% improvement over the conventional GLS algorithms.

## Introduction

Global longitudinal strain (GLS), average strain of the cardiac chamber wall measured using speckle tracking echocardiography (STE), is used for the quantification of left ventricle (LV) function. Because GLS is more robust to reader error than LV ejection fraction^1, 2^, it is increasingly used more in clinical practice^3^, especially for the detection of systolic dysfunction^4^. Moreover, the global longitudinal strain rate (GLSr), which is computed by differentiating GLS temporally^5^, quantifies the rate of LV contraction and relaxation, thus providing information on systolic and diastolic function.

Limitations of STE can impact GLS and GLSr measurement accuracy. In order to perform a GLS-STE measurement, the user typically must provide an initial shape model of the LV boundary, introducing variability and reducing measurement reproducibility^6^. Boundary tracking is subsequently performed using block-matching or cross-correlation kernels^7, 8^, sensitive to image quality, spatial and temporal resolution, and signal dropout^9^. Furthermore, commercial tools use proprietary tracking and post-processing algorithms, making cross-platform comparison impractical^6, 10^. Collectively these limitations hinder wide-spread acceptance of GLS and GLSr as diagnostic parameters with established standard ranges for normal and abnormal function^1, 4, 11–13^.

Commercial software marketed by companies including TomTec, Ultromics, and DiA provide automated strain analysis to overcome some of the above limitations. However, these software are not widely accessible. This limited access is driven by software costs and sufficient training to properly use and interpret strain outputs.

Here we present a novel algorithm for direct GLS estimation from echocardiograms that overcomes the issues mentioned above. Our approach does not require LV boundary initialization, regional smoothing, or assumptions of LV shape. The algorithm directly measures the GLSr of the entire LV, which is then integrated in time to provide the GLS. Hence, this method is robust to noise and image artifacts and minimizes user dependence by requiring only three initial feature points, one at the LV apex and two at the edges of the mitral annulus plane. Error analysis was performed using synthetic ultrasound images^14^ and clinical demonstration was performed with patient data from healthy and cardiomyopathy subjects. In both cases, we compared the results from our algorithm against conventional STE algorithms.

## Materials and methods

### Theory

During each heartbeat, the LV undergoes complex, three-dimensional motion as it contracts and relaxes. This motion is composed of a planar translation and deformation that relate how positions $\left({x}_{n},{y}_{n},{z}_{n}\right)$ along the LV move to $\left({x}_{n+1},{y}_{n+1},{z}_{n+1}\right)$^15^,

1 $$\left[\begin{array}{c}{x}_{n+1}\\ {y}_{n+1}\\ {z}_{n+1}\end{array}\right]={\varvec{F}}\left[\begin{array}{c}{x}_{n}\\ {y}_{n}\\ {z}_{n}\end{array}\right]+\overrightarrow{{\varvec{T}}}=\left[\begin{array}{ccc}{a}_{11}& {a}_{12}& {a}_{13}\\ {a}_{21}& {a}_{22}& {a}_{23}\\ {a}_{31}& {a}_{32}& {a}_{33}\end{array}\right]\left[\begin{array}{c}{x}_{n}\\ {y}_{n}\\ {z}_{n}\end{array}\right]+\left[\begin{array}{c}{t}_{1}\\ {t}_{2}\\ {t}_{3}\end{array}\right].$$

$\overrightarrow{{\varvec{T}}}$ is the translation matrix, and ***F*** is the deformation gradient tensor, which is related to the displacement gradient tensor $\nabla$ ***u***, as,

2 $${\varvec{F}}={\varvec{I}}-\nabla {\varvec{u}},$$

where ${\varvec{I}}$ is the identity matrix.

Lagrange strain, ${\boldsymbol{\varepsilon}}$, is expressed as a function of $\nabla$ ***u*** when motion and deformation are small, such that,

3 $${\boldsymbol{\varepsilon}}=\frac{1}{2}\left(\nabla {\varvec{u}}+{\nabla {\varvec{u}}}^{T}\right).$$

Equation (3) can be written as the Lagrange strain equation,

4 $$\varepsilon =\frac{l-{l}_{0}}{{l}_{0}},$$

where ${l}_{0}$ is the reference length and $l$ is the deformed length. The quantity $\varepsilon$ is the accepted definition of GLS^10^. As a result, GLS is a function of the deformation gradient tensor ***F***. In the following sections, we describe how we estimate GLS from the cross-correlation of two consecutive images.

### Pairwise Cross-Correlation

Image cross-correlation provides a statistical estimate of the translation of an image pattern between two frames. This method is used in image registration^16^, speckle tracking^17^, particle image velocimetry^18^, and image correlation^19^. The 2D discrete spatial cross-correlation between two images, ${I}_{n}$ and ${I}_{n+1}$, is expressed as,

5 $$R\left(x,y\right)=\sum_{i=-N/2}^{N/2}\sum_{j=-M/2}^{M/2}{I}_{n}\left(i,j\right){I}_{n+1}\left(x+i,y+j\right),$$

where $R\left(x,y\right)$ is the correlation plane, (*i,j*) are the summation indices of the correlation, and *N* and *M* are the image height and width, respectively. The cross-correlation can be performed in the spectral domain, as,

6 $$R\left(x,y\right)={\mathcal{F}}^{-1}\left[\overline{\mathcal{F} }\left({I}_{n}\left(x,y\right)\right)\mathcal{F}\left({I}_{n+1}\left(x,y\right)\right)\right],$$

where $\mathcal{F}$ is the 2D Fourier transformation (FT) and $\overline{\mathcal{F} }$ is the complex conjugate of the FT. The expanded form of Eq. (6) is written as,

7 $$R\left(x,y\right)=\iint {\overline{G} }_{n}\left(u,v\right){G}_{n+1}\left(u,v\right){e}^{-j2\pi \left(u\left({t}_{1}+x\right)+v\left({t}_{2}+y\right)\right)}dudv,$$

where $\left(u,v\right)$ are wavenumbers proportional to positions $\left(x,y\right)$ and $G$ is the image FT.

The Fourier transform affine theorem stipulates that rotation, stretch, and shear occurs on the FT magnitude and phase^20^. We use this to establish how the affine transform affects the rigid translation estimate by replacing *G*_*n*+*1*_ in Eq. (7) with the relationship for *G*_*n*_ using Eq. (1),

8 $$R\left(x,y\right)=\iint \frac{{\overline{G} }_{n}\left(u,v\right) {G}_{n}\left({u}^{\prime},{v}^{\prime}\right)}{\left|{\varvec{F}}\right|}{e}^{-j2\pi \left({u}^{\prime}\left({t}_{1}+x\right)+{v}^{\prime}\left({t}_{2+y}\right)\right)}dudv.$$

Here, ${u}^{\prime}={a}_{11}u+{a}_{21}v$, $v^{\prime}={a}_{12}u+{a}_{22}v$, and $\left|{\varvec{F}}\right|=det\left({\varvec{F}}\right)$. Equation (8) provides a correlation plane with the peak shifted from the plane center by $\left(\Delta \mathrm{x},\Delta \mathrm{y}\right)$, directly related to the translations ${t}_{1}$ and ${t}_{2}$ and the local deformation gradient tensor ***F***. The correlation peak shifts are written as,

9 $$\begin{array}{cc}\Delta x\cong {a}_{11}{t}_{1}+{a}_{12}{t}_{2},& \Delta y\cong \end{array}{a}_{21}{t}_{1}+{a}_{22}{t}_{2}.$$

The deformation gradients ${a}_{ij}$ produce the strain captured by the GLS measurement.

### Translation-invariant FT magnitude correlation for GLS estimation

The components of ***F*** can be estimated separately of $\overrightarrow{{\varvec{T}}}$ using the magnitude, $\left|G\left(u,v\right)\right|$, of the FT^20, 21^. The cross-correlation of the FT magnitudes is *translation invariant* yielding the terms of ***F*** with no contribution from $\overrightarrow{{\varvec{T}}}$. The *log-polar* basis Fourier-Mellin transform^22, 23^, popular in image registration, decouples terms of ***F*** to estimate image rotation and stretch.

Contraction and relaxation of the LV result in deformation akin to anisotropic image rescaling. By changing the FT magnitude image coordinates from cartesian $\left(u,v\right)$ to orthogonal logarithmic coordinates $\left(logu,logv\right)$, the resulting displacements from the correlation between two FT magnitude images now correspond to horizontal $\Delta u$ and vertical $\Delta u$ rescaling such that,

10 $$|G\left(logu+log\Delta u,logv+log\Delta v\right)|.$$

This correlation is affected by rotation and shear based on the terms present in Eq. (8). However, if these terms are minimized beforehand, the correlation peak shift estimates $\left(\Delta u,\Delta v\right)$ can be related to the terms ${a}_{11}$ and ${a}_{22}$ from ***F*** through,

11 $$\begin{array}{cc}{a}_{11}=\Delta u\cong {e}^{\Delta x},& {a}_{22}=\Delta v\cong {e}^{\Delta y}.\end{array}$$

We substitute ${a}_{11}$ and ${a}_{22}$ from Eq. (11) into ***F*** in Eq. (2) and solve for $\nabla \mathbf{u}$ such that,

12 $$\nabla \mathbf{u}=\left[\begin{array}{cc}{e}^{dx}-1& 0\\ 0& {e}^{dy}-1\end{array}\right].$$

Since deformation of the LV in long axis apical (ALAX) scans occurs along its length, we assume GLS occurs predominantly along the vertical direction, providing the GLS estimator,

13 $${\varepsilon }_{GLS}=\frac{l-{l}_{0}}{{l}_{0}}\approx {e}^{dy}-1.$$

### Direct global longitudinal strain estimation algorithm

**Figure 1:**
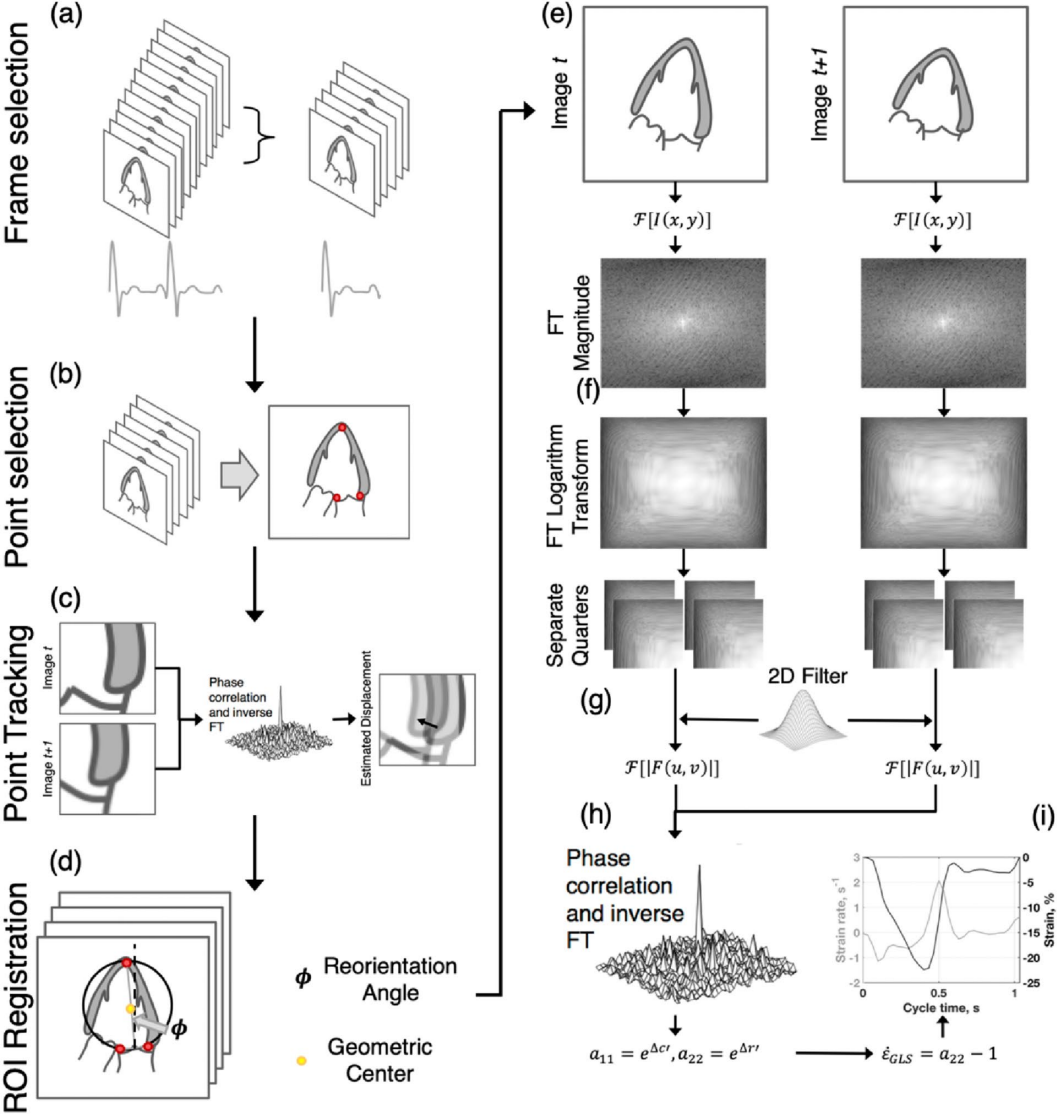
Illustration of the direct global longitudinal strain estimation algorithm. (**a**) Frames for a single beat are selected from an input echocardiogram. (**b**) User inputs for the apex and annulus positions from a reference frame are provided. (**c**) The inputs are tracked temporally. (**d**) Frame co-registration is performed. (**e**) The LV is cropped from each frame, and these sub-images are Fourier-transformed. (**f**) The FT magnitude is calculated, interpolated onto a logarithm-basis, and separated into four sub-images. (**g**) Each sub-image is Fourier-transformed and convolved with a phase filter. (**h**) Ensemble phase correlation is performed, producing a correlation plane with a peak shifted from the plane center. This shift corresponds to a frame pair strain rate. (**i**) Strain is computed by temporally integrating the strain rate estimates.

We now describe our algorithm using the translation-invariant FT magnitude correlation to estimate the GLS based on Eq. (13). A schematic of our algorithm is provided in Fig. 1. The algorithm comprises two stages—the first performs an image registration to minimize shear and rotation that corrupt the correlation accuracy, while the second performs the GLS estimation.

The algorithm begins by selecting frames to analyze (Fig. 1a). Next, the user selects three points from the first frame (Fig. 1b), corresponding to approximate locations of the LV apex, the annulus septal, and annulus lateral positions. These points are tracked between consecutive frames using standard pairwise cross-correlation (Fig. 1c). For each frame, the geometric center from the tracked points is computed along with the vertical axis orientation angle for a line formed from the annulus center to the apex (Fig. 1d). Frames are then aligned based on the geometric center, and the orientation angle is corrected. A circular ROI for each frame is defined from the tracked points. This ROI is applied to filter out most of the tissue signal present from the right ventricle (RV) free wall and left atrium (LA).

In the second stage, for each pair of sequential registered images, *t* and *t* + *1* (Fig. 1e), their FT and FT magnitude is computed. The FT magnitudes are interpolated from the image grid onto a logarithm-scale grid (Fig. 1f). Because the FT logarithm transformed images are symmetric about the image diagonals, they are separated into four quarters to improve measurement accuracy. Each sub-image is filtered^24^ to further minimize the influence of tissue signal that may remain from the RV free wall and LA, preserving the signal content from the LV septal wall and LV free wall, and their FT is computed (Fig. 1g). The FT sub-images are then correlated using the *spectral* cross-correlation kernel and ensemble-averaged to provide the displacements $\left(\Delta \mathrm{x},\Delta \mathrm{y}\right)$ (Fig. 1h). A dynamic phase-filtered kernel is applied to the cross-correlation to improve estimate accuracy^25–27^. The displacements $\left(\Delta \mathrm{x}, \Delta \mathrm{y}\right)$ are adjusted based on the logarithm-scale grid, becoming $\left(\Delta \mathrm{x^{\prime}},\Delta \mathrm{y^{\prime}}\right)$. The GLSr between frames is computed using $\Delta{\mathrm{y}}^{\prime}$ and Eq. (13). Finally, GLSr across each frame pair is integrated in time using 4th-Order Runge–Kutta to obtain GLS (Fig. 1i). The integral operator provides inherent smoothing, which suppresses noise in the GLS measurements. Drift correction is applied to ensure the measurement returns to an undeformed state. We will hereafter refer to this method as the Logarithm-Transform Correlation (LTC) method.

### Speckle tracking strain

This study uses two standard STE algorithms against which we benchmark our method. One algorithm uses the spatial cross-correlation kernel introduced in Eq. (5)^17^, referred to herein as the *Direct Cross-Correlation* or DCC method. The second uses the spectral cross-correlation (Eq. 6), hereafter referred to as the Fourier Transform Correlation or FTC method.

Boundary tracking is performed by propagating the segmented boundary of the initial frame through the estimated displacement fields using 4th-Order Runge–Kutta. GLS is estimated as the measured change in arc-length between the segmented and the tracked boundary from each frame. Image co-registration is not performed, as it is not required to obtain a consistent GLS measurement based on the arc-length change calculation. Drift correction is applied to ensure the measurement returns to an undeformed state.

### Artificial echocardiograms

Error analysis was performed using synthetic LV ALAX echocardiograms generated by and made publicly available from the Laboratory on Cardiovascular Imaging and Dynamics at KU Leuven^14, 28^. The synthetic echocardiograms mimic images acquired from Genera Electrics (GE) Vivid E9, Hitachi-Aloka Prosound $\alpha$7 CV, Philips iE 33, Siemens SC2000, and Toshiba Artida vendor machines. The datasets provide error sources from vendor realistic noise and tissue speckle signal loss due to out of plane motion^14^. Frame rate was varied to match the specific vendor machine. Two-chamber (A2C), three-chamber (A3C), and four-chamber (A4C) LV ALAX views were provided in the dataset for one normal and four ischemic conditions. The specific occlusions include distal and proximal left anterior descending artery, left circumflex artery, and right coronary artery. Ground truth boundaries, displacements, and strains for each dataset were included with the synthetic images.

Mean absolute error (MAE) and root-mean-square error (RMSE) for GLS and GLSr were quantified as a function of contrast to noise ratio (CNR) for all LV ALAX views under all ischemic or normal conditions. A GLS and GLSr measurement was obtained for each of the frames in every echocardiogram analyzed. A total of 4,380 measurements were examined for each of the different measurement methods. The MAE and RMSE were calculated based on a ground truth GLS value for each frame which was quantified from the average segmental strain around the LV boundary using the first frame in each echocardiogram as the reference or zero-strain frame.

CNR was defined as the ratio between the difference of the means and the variance between the tissue signal and the signal inside the LV^29^,

14 $$CNR= \frac{\left|{\mu }_{V}-{\mu }_{T}\right|}{\sqrt{{\sigma }_{V}^{2}+{\sigma }_{T}^{2}}} ,$$

where ${\mu }_{V}$ and ${\mu }_{T}$ are the mean of the signal intensity inside the ventricle and throughout the myocardial tissue, respectively, and ${\sigma }_{V}$ and ${\sigma }_{T}$ are the standard deviation of the signal inside the ventricle and through the myocardial tissue, respectively. Boundaries for the ventricle and myocardium are included with the ground truth data and were used here to perform the CNR calculation for each frame in the artificial echocardiograms.

Error quantities were normalized by the peak GLS or peak GLSr.

### Clinical imaging

The method's clinical capabilities were demonstrated using a cohort of pediatric patients with confirmed cardiomyopathy and age-matched controls collected from a study conducted at the University of Nebraska Medical Center in Omaha, Nebraska, USA. The Institutional Review Boards of Purdue, Nebraska, and Johns Hopkins Universities each approved the study protocol. All procedures were performed in accordance with relevant guidelines and regulations. Informed written consent was obtained from study subject guardians for those under age 18 or from the subject themself for those over age 18.

Each patient underwent a routine echocardiogram study on an iE33 ultrasound system (Philips Healthcare, Andover, MA, USA). Studies were collected based on the American Society of Echocardiography recommendations^30^. The 53-subject cohort included 4 patients with confirmed dilated cardiomyopathy (DCM), 16 patients with confirmed hypertrophic cardiomyopathy (HCM), and 33 age-matched controls. Information on the cohort demographics is provided in Table 1 and heart function indices in Table 2.

**Table 1:** Demographics of the study cohort for each disease state.

Characteristics	Control (*n* = *33*)	DCM (*n* = *4*)	HCM (*n* = *16*)	*p*
Age (years)	17.98 ± 8.86	14.50 ± 6.24	18.74 ± 10.47	0.718
BSA (m^2^)	1.66 ± 0.56	1.52 ± 0.64	1.81 ± 0.69	0.592
Height (cm)	159.25 ± 29.11	147.90 ± 57.60	159.81 ± 30.86	0.766
Weight (kg)	63.50 ± 30.42	57.60 ± 35.65	75.71 ± 41.03	0.436
Heart rate (bpm)	67.47 ± 17.26	92.50 ± 33.81	72.88 ± 18.78	0.057

**Table 2:** Indices for LV dimensions and functional parameters.

	Control (*n* = *33*)	DCM (*n* = *4*)	HCM (*n* = *16*)	*p*
**Ventricular dimensions**				
End diastolic volume (ml)	98.85 ± 39.19	178.75 ± 83.92	96.28 ± 37.66	0.003
End systolic volume (ml)	37.80 ± 15.84	117.75 ± 60.31	36.03 ± 18.40	< 0.001
Stroke volume (ml)	61.29 ± 24.39	61.00 ± 33.32	59.44 ± 21.24	0.983
Ejection fraction (%)	62.16 ± 3.50	34.25 ± 14.93	63.06 ± 6.01	< 0.001
**Functional parameters**				
E-wave velocity (cm s^−1^)	82.20 ± 19.70	102.25 ± 29.80	83.19 ± 19.59	0.201
A-wave velocity (cm s^−1^)	42.84 ± 10.95	61.25 ± 34.74	61.13 ± 33.65	0.023
e’ velocity (cm s^−1^)	17.58 ± 3.22	11.38 ± 2.63	10.51 ± 3.04	< 0.001
E/A ratio	2.00 ± 0.59	2.24 ± 1.62	1.55 ± 0.50	0.061
E/e’ ratio	4.06 ± 1.21	8.22 ± 4.30	7.34 ± 2.58	< 0.001

Doppler measurements were collected in the ALAX A4C view. B-mode ALAX A2C, A3C, and A4C view scans were performed conventionally, not explicitly collected for strain measurements. B-mode scan frame rates varied between 29 frames per second (FPS) and 100 FPS, with a median of 50 FPS. Images were stored in Digital Imaging and Communications in Medicine (DICOM) format for post-processing. Ventricle dimension measurements were computed by the Simpson biplane method using the GE EchoPAC software. Data were classified into control (CTRL) and cardiomyopathy (CM) groups.

Peak absolute GLS (GLS_P_) and peak absolute systolic GLSr (GLSrs) were computed for each patient across all ALAX views. When appropriate, repeated measurements within each ALAX view were averaged. GLS_P_ and GLSrs are reported as the average of the three standard ALAX views^6^. Patient data was similarly evaluated using commercially available software (Image-Arena Version 4.6 Build 4.6.2.12, TomTec, Germany). Only one scan from each ALAX view was analyzed per Nebraska’s standard clinical practices. Statistical significance was tested by one-way analysis of variance (ANOVA) and Tukey Honestly Significant Difference (HSD). Receiver operating characteristic (ROC) curves and area under the curve (AUC) were computed by repeated measurement random sampling from each view to report curves with 95% confidence intervals. The ROC performance was characterized by quantifying the Youden Index and distance to corner parameters. The Youden Index (YI) is a measure of informedness, or how well informed a classification test would be based on predictions, defined as

15 $$YI=sensitivity+specificity-1 ,$$

with a range of 0 to 1, where 0 is no predictive ability and 1 is perfect predictive ability^31^. Distance to corner is a measure of the ROC curve at its optimal threshold to a perfect prediction at a sensitivity of 1, or perfectly predicting true positive class, and 1-specificity of 0, or perfectly predicting the true negative class.

## Results

### Error analysis results

**Figure 2:**
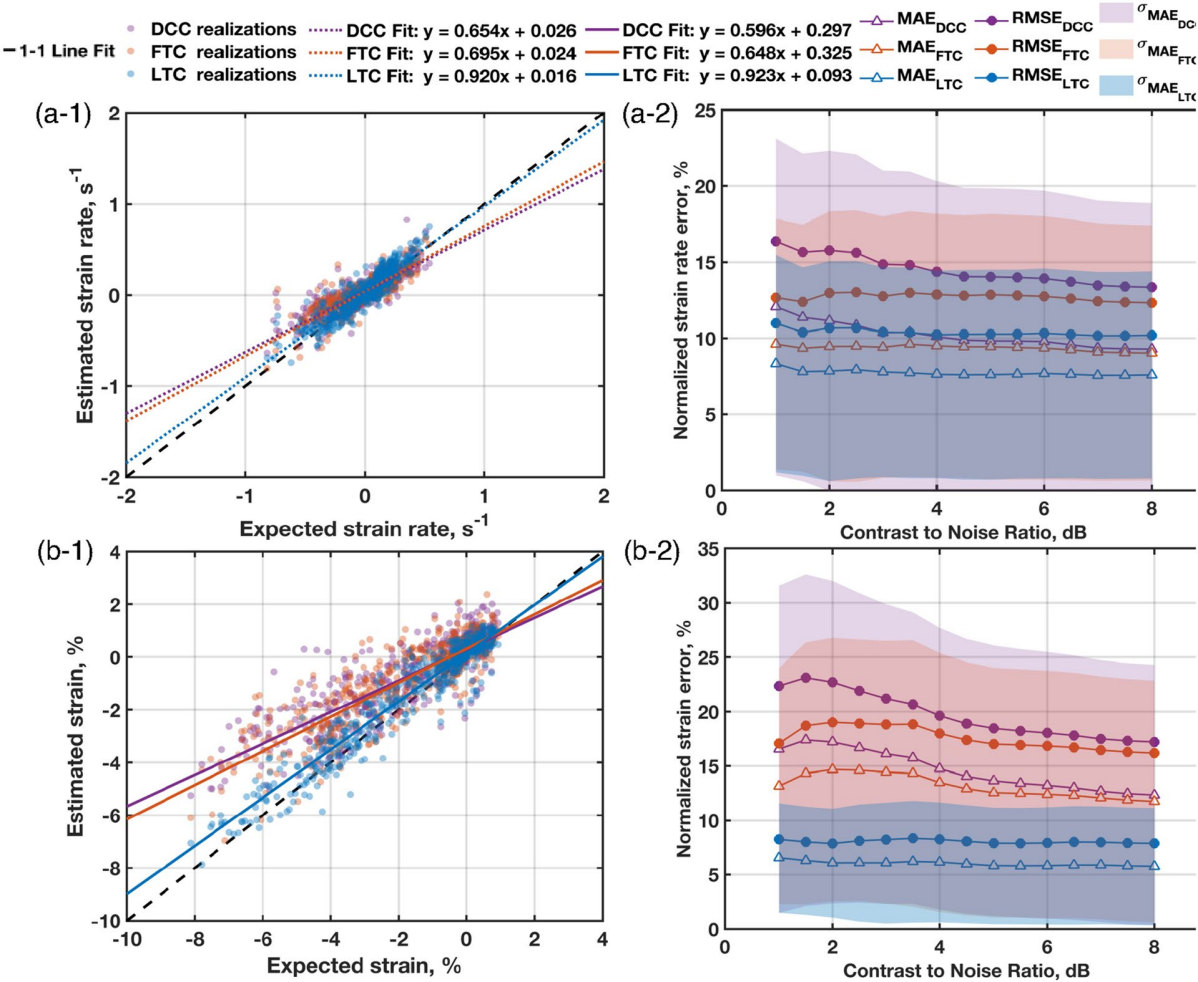
(Left) Direct comparison of measurements to ground truth values and (right) normalized mean absolute error (MAE) and root mean square error (RMSE) as a function of contrast-to-noise ratio (CNR) for (**a**) GLSr and (**b**) GLS quantities. Measurements were performed using the Direct Cross-Correlation method (DCC), Fourier Transform Correlation (FTC), and Fourier-based Logarithm Transform Correlation (LTC). Standard deviation of the absolute error measurements are provided as the shaded regions around the MAE curves.

Figure 2 presents error analysis results. Error as a function of CNR was binned from 1 to 8 dB in 0.5 dB increments. Each bin spanned a range of 0.5 dB. Marker bin sizes varied from a minimum of 285 measurements to a maximum of 1260 measurements. Each marker represents the average MAE or RMSE of the error that fall within each CNR bin. The shaded regions on the plot represent the standard deviation of the absolute error measurements that fall within each CNR bin.

The GLSr estimates (Fig. 2a-1) yielded a linear regression fit with slope and bias of ${m}_{DCC}$ = $0.65\mathrm{s}\cdot {s}^{-1}$ and ${b}_{DCC}=0.03{ s}^{-1}$ for the DCC method, ${m}_{FTC}$ = $0.70{\mathrm{s}}\cdot {s}^{-1}$ and ${b}_{FTC}=0.02{ s}^{-1}$ for the FTC method, and ${m}_{LTC}=0.92{\mathrm{s}}\cdot {s}^{-1}$ and ${b}_{LTC}=0.02 {s}^{-1}$ for the LTC method. Regression fit qualities of ${R}_{DCC}^{2}=0.72$, ${R}_{FTC}^{2}=0.76$, and ${R}_{LTC}^{2}=0.85$ were measured. GLSr, as a function of CNR, is shown in Fig. 2a-2 (values are normalized by $GLSr=0.95{ s}^{-1}$). LTC showed a 1.5 to 2-fold improvement in accuracy than DCC and a 1.5-fold improvement compared to FTC. Additionally, the LTC method is unaffected by CNR. In contrast, the FTC and DCC methods show a CNR dependence, albeit less for the FTC method.

GLS estimates (Fig. 2b-1) yielded a linear regression fit with slope and bias of ${m}_{DCC}$=$0.60$ and ${b}_{DCC}=0.30\mathrm{\%}$ for the DCC method, ${m}_{FTC}$=$0.65$ and ${b}_{FTC}=0.33\mathrm{\%}$ for the FTC method, and ${m}_{LTC}=0.92$, ${b}_{LTC}=0.09\mathrm{\%}$ for the LTC method. Regression fit qualities of ${R}_{DCC}^{2}=0.71$, ${R}_{FTC}^{2}=0.74$, and ${R}_{LTC}^{2}=0.91$ were measured. The LTC method shows more than 200% improvement in measurement accuracy compared against the DCC and FTC methods as a function of CNR (Fig. 2b-2), where values are normalized by $GLS=8.47\%.$ The error analysis demonstrates that the LTC method is unaffected by CNR, while the DCC and FTC methods are affected by signal quality.

### Clinical analysis results

**Figure 3:**
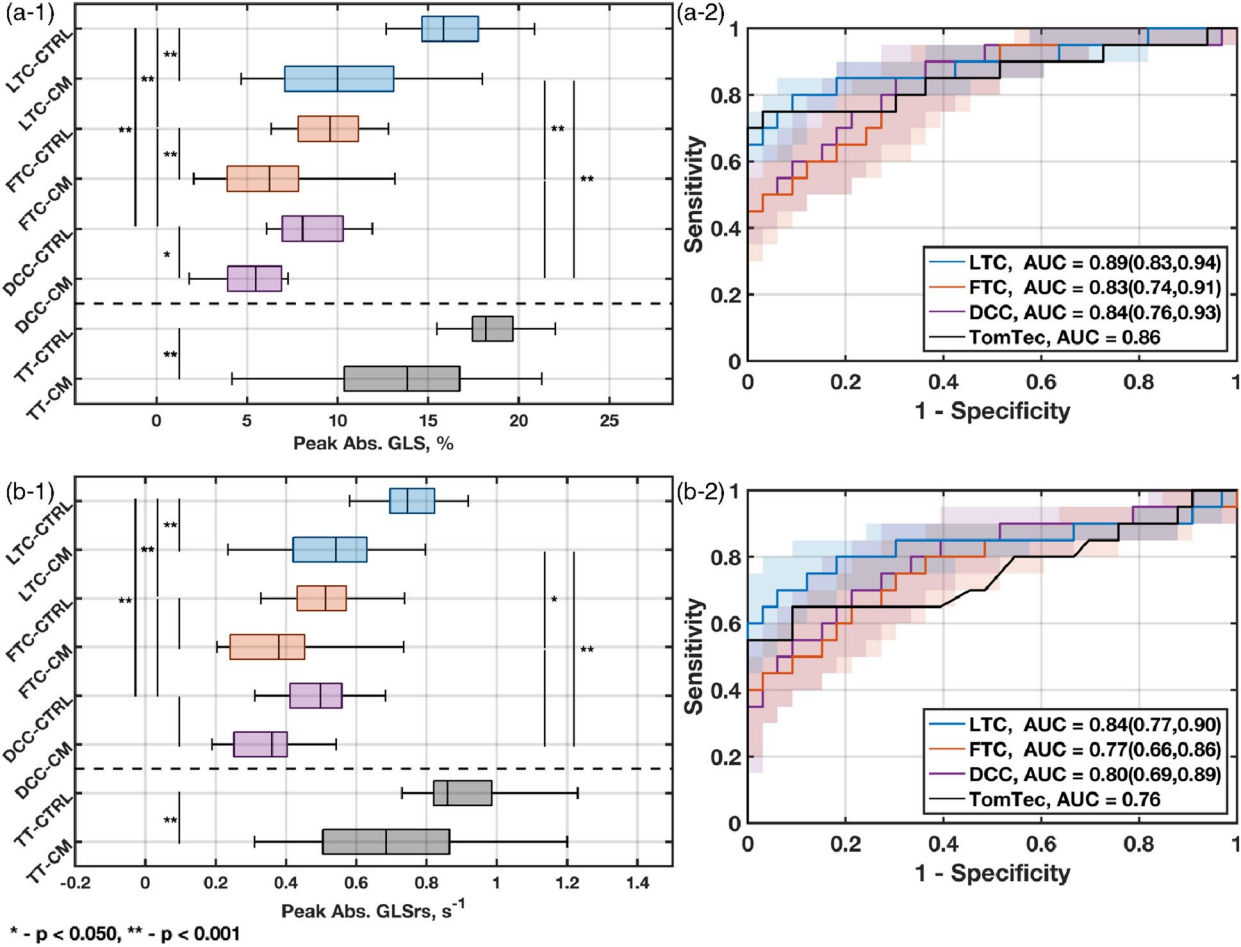
Distribution of measurements and significance tests for each GLS measurement method on observing (**a-1**) peak absolute GLS (GLS_P_) and (**b-1**) peak absolute systolic GLSr (GLSrs). (2) Receiver operating characteristic (ROC) curves displaying the ability for the LTC method estimated parameters to distinguish between normal and abnormal cardiac disease states based on (**a-2**) GLS_P_ and (**b-2**) GLSrs. The analysis was performed on a set of control subjects (CTRL) and subjects with cardiomyopathies (CM). The LTC method was compared with the conventional STE methods, FTC and DCC, as well as with the commercially available TomTec software. The bounded regions about the ROC curve provide the 95% confidence interval over which sensitivity and specificity are measured, contingent on the beat records and scans analyzed within subjects.

Results comparing the LTC method against conventional GLS methods and TomTec are presented in Fig. 3a-1,b-1. The LTC median GLS_P_ are higher compared to the conventional methods (GLS_P,LTC-CTRL_ = $$15.84\%$$, GLS_P,LTC-CM_ = $$10.00\%$$; GLS_P,FTC-CTRL_ = $$9.59\%$$, GLS_P,FTC-CM_ = $$6.25\%$$; GLS_P,DCC-CTRL_ = $$8.07\%$$, GLS_P,DCC-CM_ = $$5.47\%$$), but lower compared to TomTec (GLS_P,TT-CTRL_ = $$18.19\%$$, GLS_P,TT-CM_ = $$13.86\%$$). Significance tests by ANOVA indicated the GLS_P_ group means are significant (F-value = 90.02; $$p<1\times {10}^{-5}$$). Post-hoc analysis by Tukey HSD indicated the group means between health states were significant ($$p<0.05)$$ for all methods. Furthermore, the LTC method was statically significant ($$p<0.05)$$ compared to the conventional methods for both health states. Differences were not statistically significant between the FTC and DCC methods.

Similarly, for the GLSrs measurements, the LTC medians are higher than compared to the conventional methods (GLSrs_LTC-CTRL_ = $$0.75{s}^{-1}$$, GLSrs_,LTC-CM_ = $$0.54{s}^{-1}$$; GLSrs_FTC-CTRL_ = $$0.51{s}^{-1}$$, GLSrs_,FTC-CM_ = $$0.38{s}^{-1}$$; GLSrs_LTC-CTRL_ = $$0.{50s}^{-1}$$, GLSrs_,LTC-CM_ = $$0.36{s}^{-1}$$) but lower compared to TomTec (GLSrs_LTC-CTRL_ = $$0.86{s}^{-1}$$, GLSrs_,LTC-CM_ = $$0.69{s}^{-1}$$). Significance tests by ANOVA indicated the GLSrs group means are significant (F-value = 44.35; $$p<1\times {10}^{-5}$$). Tukey HSD post-hoc analysis indicated the group means between health states were significant ($$p<0.05)$$ for the LTC and TomTec methods, but were not significant for the conventional methods. Moreover, the LTC method was statically significant ($$p<0.05)$$ compared to the conventional methods for both health states. Results were not significant between the FTC and DCC methods.

ROC curves are presented in Fig. 3a-2, b-2. The GLS_P_ ROC curves (Fig. 3a-2) show AUCROC values of GLS_P,LTC-AUCROC_ = $0.89$; GLS_P,FTC-AUCROC_ = $0.83$; GLS_P,DCC-AUCROC_ = $0.84$; and GLS_P,TT-AUCROC_ = $0.86$. The LTC ROC confidence interval indicated this method at the low end performed as well as TomTec when classifying patients, but could improve classification by 10% based on the beats analysed. The FTC and DCC methods show increased confidence intervals, but consistently performs worse than the LTC method. The LTC ROC performance was marginally better than the other methods based on performance metrics (YI_LTC_ = $0.79$, CD_LTC_ = $0.22$; YI_LTC_ = $0.42$, CD_LTC_ = $0.36$; YI_DCC_ = $0.54$, CD_DCC_ = $033$; YI_TT_ = $0.72$, CD_TT_ = $0.25$).

The GLSrs ROC curves (Fig. 3 b-2) show the LTC AUCROC is consistently higher compared to the other methods (GLSrs_LTC-AUCROC_ = $0.84$; GLSrs_FTC-AUCROC_ = $0.77$; GLSrs_DCC-AUCROC_ = $0.80$; GLSrs_TT-AUCROC_ = $0.76$). The confidence interval for the LTC method was reduced compared to the conventional strain methods and shows at the low end that LTC is comparable to TomTec but can offer a 15% improvement overall. Additionally, the LTC ROC performance was better than the other methods based on performance metrics (YI_LTC_ = $0.62$, CD_LTC_ = $0.27$; YI_LTC_ = $0.45$, CD_LTC_ = $0.39$; YI_DCC_ = $0.49$, CD_DCC_ = $037$; YI_TT_ = $0.56$, CD_TT_ = $0.36$).

## Discussion

This study presents a new algorithm, the LTC method, for computing GLS and GLSr estimates from ultrasound scans. Error analysis using synthetic ultrasound images quantified the LTC method's improvement over two conventional STE methods. A clinical cohort was analyzed using the LTC method, the two conventional STE methods, and a commercial software method. The LTC method does not rely on LV shape assumptions and avoids the use of boundary segmentation. Furthermore, because LV segmentation is not required, regularization to preserve the segmentation shape is avoided. Moreover, the entire image of the LV is used to compute strain which minimizes out-of-plane motion correlation loss.

These claims are supported by basic principles derived in particle image velocimetry (PIV), another FFT-based cross correlation application. In PIV, fluid tracing particle motion is measured between frames^32^. Out-of-plane motion in PIV images, which are speckle-like in nature, is a function of sample volume and out-of-plane velocity gradients^33^. PIV frame rates are high (greater than 100 FPS) and the illumination volume is thin (approximately 1 mm). In B-mode imaging, the sample volume is thicker (upwards of 10 mm) and frame rates are sufficiently high (more than 30 FPS) relative to cardiac tissue velocity gradients (on the order of 100 mm/s, or roughly 3 mm/frame at the lowest frame rate settings). Thus, speckle pattern losses in the LTC should not be significant, more so with additional signal content provided from the whole LV image.

The LTC method is novel by directly computing the GLSr between sequential frames, ensuring a reliable rate measurement. Computing GLS by integrating the GLSr provides a smoothing operation that reduces noise. Commercial GLS algorithms have constraints that enforce tracked boundaries that are smooth in space and time, providing measurements that appear physically consistent, but become a function of the regularization process^10, 34, 35^. Thus, the GLS measurements do not adhere to the underlying deformation that occurs between frames.

The error analysis presented in Fig. 2 demonstrated that the DCC and FTC methods are dependent on CNR. As CNR approaches 1, the mean pixel intensity of the LV wall and the mean pixel intensity of speckle noise are almost equal. This means the speckle noise cannot be differentiated from the LV wall, making the physical features ambiguous. Both methods underestimated the GLS, supporting the notion that it may be best to avoid such computation^6^. In contrast, the LTC method enables robust GLS computation even with noisy images, as supported by the MAE and RMSE plots in Fig. 2.

**Figure 4:**
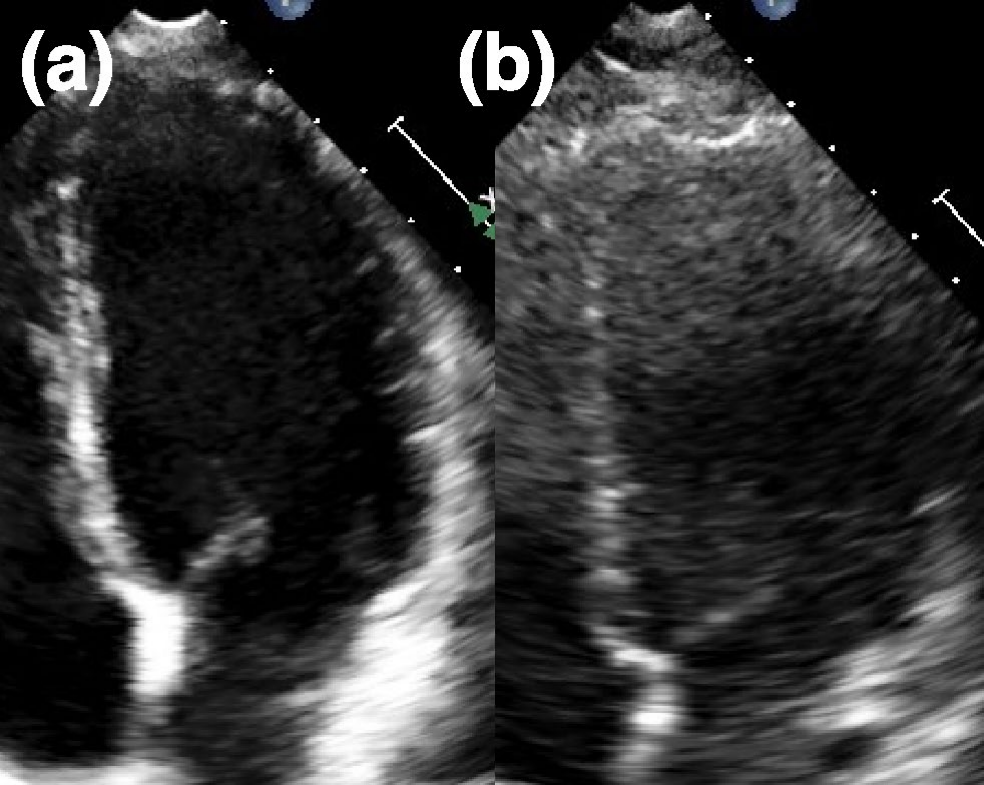
Demonstration of contrast-to-noise (CNR) differences for echocardiogram scans from clinical test subjects. (**a**) High CNR (> 8 dB), where there is little signal within the LV and strong tissue signal in the myocardium, composing the LV. (**b**) Low CNR (< 2 dB), where there is high, incoherent speckle noise within the LV and weak tissue signal along with signal loss within the myocardium composing the LV. These images were taken from two female subjects, one aged 21 years old (y/o) and the other aged 24 y/o.

For demonstration purposes, example images from two test subjects’ clinical data are provided to show the quality differences that affect CNR, provided in Fig. 4. These subjects are of the same gender and similar ages. In, one scan (Fig. 4a) the LV interior is clear of noise and the myocardium can be clearly delineated, providing a CNR of greater than 8 dB. The other scan (Fig. 4b) has increased noise within the LV interior and signal loss in the LV myocardium, resulting in a CNR of less than 2 dB. These differences for the latter case can greatly impact conventional STE method measurement accuracy.

Comparison of each method for all health states, presented in Fig. 3a-1,b-1, provides the measurement distributions and their statistical significance. For each method, the variance of the CTRL and CM distributions overlapped while statistical significance of the means was observed. Between methods, statistical significance was observed for the LTC method compared to the conventional STE methods. These results indicate larger GLS_P_ and GLSrs values for normal function than for abnormal function, but establishing improvement of the LTC method through this analysis alone is not possible.

The ROC curves, presented in Fig. 3a-2,b-2, are used to determine if clinical separation is possible. The LTC method GLS_P_ ROC curve showed marginal classification improvement compared to the conventional STE methods and the commercially available method. However, GLSrs ROC curve shows a nearly 10% improvement in classification. Both tested parameters show improved correct diagnosis rates.

The LTC method GLS_P_ and GLSrs measurements were below nominal ranges reported from literature (peak GLS > 18%; peak GLSrs > 1 s^−1^)^36^. Commercial methods rely on regularization steps, which force the measurements to fit in LV shape models^6, 10^, thereby causing the GLS measurements to be a function of the regularization instead of the actual underlying deformation, possibly leading to overestimation^37, 38^.

LTC algorithm limitations stem from the assumption that GLS and GLSr can be reliably measured from the septal and lateral walls, ignoring shortening near the apex and possible effects of signal loss, attenuation, and phase aberration along the myocardium. Furthermore, the algorithm assumes that reorientation has been performed correctly with no misalignment. If misalignment exists, the measurements will be a combination of GLS and off-axis strain. Out-of-plane motion in the artificial dataset is modelled, but may cause speckle signal dropout, requiring a correction step to mitigate gaps in the images^14^. Thus, a correction step is used which may minimize out-of-plane motion and reduce influence of this error component. Frame rate is also known to affect strain measurement accuracy, with a typical operating range of 50–80 FPS^9, 10^. The LTC method should be evaluated in future work to determine if this optimal range is suitable for application or if lower or higher frame rates must be maintained for accurate measurements. Robustness of the LTC correlation to noise and out-of-plane correlation loss should be tested in greater detail in a future study, however this does require rigorous and controlled test conditions which is not trivial. Finally, this study was limited by its clinical validation, which was performed using retrospective pediatric data. Pediatric echocardiograms are typically of good quality, without significant attenuation, clutter noise, or phase aberration which are common in adult echocardiograms for patients with cardiovascular disease. A dataset optimized for GLS estimation would help further substantiate findings. Future work will apply the LTC algorithm to study patient populations with ischemic heart disease and cardiotoxicity-related heart failure.

We presented a new correlation kernel, the logarithm transform correlation or LTC, for quantifying GLS and GLSr from echocardiography scans. Our LTC-based algorithm does not use LV shape assumptions, is machine-agnostic, automated, and free of heuristic inputs. We compared the LTC against STE algorithms using artificial scans, analyzing error against ground truth GLS and GLSr values, and validated using clinical data from a study of pediatric cardiomyopathies. Results showed that the LTC method is unaffected by the image quality, providing improved measurement accuracy against the STE methods for both the synthetic data and clinical cohort.
